# Prevalence and risk factors for pelvic floor disorders during early and late pregnancy in a cohort of Austrian women

**DOI:** 10.1007/s00404-019-05311-9

**Published:** 2019-10-10

**Authors:** Barbara Bodner-Adler, Oliver Kimberger, Thomas Laml, Ksenia Halpern, Clara Beitl, Wolfgang Umek, Klaus Bodner

**Affiliations:** 1 Department of General Gynecology and Gynecologic Oncology, Medical University of Vienna, Währinger Gürtel 18-20, Vienna, 1090 Austria; 2 Department of Special Gynecology and Obstetrics, Karl Landsteiner Institute, Vienna, Austria; 3 Department of Anesthesiology, Medical University of Vienna, Vienna, Austria; 4 Outcomes Research Consortium, Cleveland, OH USA; 5 Department of Obstetrics and Fetomaternal Medicine, Medical University of Vienna, Vienna, Austria; 6 Department of Gynecology and Obstetrics, General Hospital of Rudolfstiftung, Vienna, Austria

**Keywords:** Prevalence, Risk factors, Pelvic floor-related quality of life, Modified pelvic floor questionnaire, Pelvic floor disorders, Pregnancy

## Abstract

**Purpose:**

To investigate the prevalence of pelvic floor disorders (PFDs) in a cohort of Austrian women either during their early or late pregnancy and to search for clinical risk factors which correlate with pelvic floor symptoms during pregnancy.

**Methods:**

A prospective study was conducted and 200 pregnant women answered the validated German pelvic floor questionnaire during their first or third trimenon of gestation. Furthermore, a multivariate logistic regression model was used to determine independent risk factors for PFDs after adjusting for confounders.

**Results:**

96/200 (48%) women reported psychological strain in at least 1 of the 4 pelvic floor domains while the remaining 104 women (52%) were asymptomatic. Affected women showed a significant higher BMI, a more frequent positive family history and a higher rate of multiple pregnancies was noted compared to asymptomatic women (*p* < 0.05). Furthermore, a statistically significant positive correlation could be observed between BMI, smoking and mean bladder score as well as mean prolapse score, signifying more symptom bother from bladder and prolapse in smokers with high BMI. A significant positive correlation was also detected between mean bowel score and parity. In the multivariate model, high BMI (CI 1.013–1.143), positive family history (CI 0.044–0.260) and multiple pregnancies (CI 0.011–0.244) remained independently associated with pelvic floor symptoms (*p* < 0.05).

**Conclusion:**

Our results demonstrate that pelvic floor-related quality of life during pregnancy is a prevalent condition which is strongly affected by the expectant mother’s weight as well as her family history. In addition, women with multiple pregnancies seem to be at increased risk.

## Introduction

Pelvic floor disorders (PFDs) can adversely affect the quality of life (QoL) of a woman and they can occur during different stages of female life such as during pregnancy, early postpartum period or during menopause [1]. The reported prevalence of PFDs varies widely both during and after pregnancy [1–3] with SUI rates, e.g., up to 30–50% [4, 5]. Besides, the term PFD includes a broad spectrum of conditions such as urinary incontinence (UI), pelvic organ prolapse (POP) or anal incontinence (AI) [1]. Many risk factors seem to be involved like the pregnancy itself, mode of delivery, parity, the use of episiotomy, obesity, increased age and so on [2, 4–6]. It is well known that pregnancy and vaginal birth are significant risk factors in the etiology of PFDs [7] and predicting models like UR-CHOICE score were developed for this reason to provide mothers-to-be with sufficient information regarding their subsequent risk of PFD [8, 9]. The identification of women during their pregnancy who are at higher risk for PFD remains a key element in targeting of prevention and planning health of resource allocation strategies.

Pregnancy, childbirth and the immediate postpartum period, where the demands on the pelvic floor and the incidence of pelvic floor trauma are particularly high, offers an optimal opportunity for such counseling and prevention. The validated German pelvic floor questionnaire modified for pregnancy and postpartum period is thus an important tool identifying such symptoms and helping clinicians assess patient’s quality of life [10]. The aim of the present study was to evaluate the prevalence of PFDs in a cohort of Austrian women either during early (first trimester) or late (third trimester) pregnancy. Furthermore, we searched for clinical risk factors which correlate with the occurrence of PFDs during pregnancy in our population.

## Materials and methods

### Study population

This prospective study was conducted between September 2018 and June 2019 at the department of obstetrics and fetomaternal medicine of the Medical University of Vienna (MUVI), which is the main tertiary center in the region with an annual birth rate of around 2800 deliveries/year. During the study period, eligible patients received the German version of the modified validated pelvic floor questionnaire (return rate: 99% in all) to investigate the prevalence of PFDs in a cohort of Austrian women during their early or late weeks of pregnancy (either first or third trimester). The main outcome variable of interest was subjective pelvic floor-related quality of life symptoms. The rationale of the study was clearly explained. Informed consent was obtained prior to completion of the self-administered questionnaire and the study protocol gained ethical approval from the ethics committee of MUVI (EK No.17447/2018).

Women were recruited from the maternity outpatient clinic and inclusion criteria included age over 18 years, first or third trimester of pregnancy with planned delivery at our institution. Women with inability to complete the questionnaire due to a language problem were excluded from the study.

Women ran through the questionnaire either during their first or last visit at the outpatient clinic and afterwards they were classified into two groups: patients with one or more PFDs (*n* = 96/200) (= significant psychological strain in at least one pelvic floor domain) and patients without any pelvic floor complaints (*n* = 104/200). Clinical information, including obstetrical and neonatal data were obtained from the database of the department of obstetrics and fetomaternal medicine (PIA software). All patient records were anonymized and de-identified prior to analysis.

### Comprehensive pelvic floor questionnaire during pregnancy and postpartum period

The modified German pelvic floor questionnaire is a self-administered, validated questionnaire for the assessment of pelvic floor disorders, their risk factors and their impact of quality of life during pregnancy and postpartum period which integrates bladder, bowel and sexual function, pelvic organ prolapse, severity, bothersomeness and condition-specific quality of life in women with urinary incontinence (UI) and/or POP. The questionnaire is divided into four main domains (bladder, bowel, pelvic organ prolapse, sexual function) and each question is scored from zero to three. The additive scores are divided by the maximum reachable score and multiplied by ten, giving a value between zero (0 = no symptoms) and ten (10 = maximum symptoms) for each of the domains. Results of the validation study and scoring system have been published previously by Baessler et al. [10].

### Statistical analysis

Chi-square was used for the comparison of categorical variables between the two groups and Student’s *t* test for continuous variables. The average score of each domain in the questionnaire was reported as mean and standard deviation. For correlation analysis, Spearman test was used with correlation coefficient. Multivariate stepwise logistic regression (including backward elimination) was performed to identify parameters associated with pelvic floor disorders. A *p* value < 0.05 was considered statistically significant. The SPSS system (IBM, Armonk, NY, USA, Version 23) was used for the calculations.

## Results

Two hundred and nine (209) women in all gave consent to participate in this study, 200 of whom (96%) were finally included after returning an evaluable complete survey. Missing data did not exceed 4%. Clinical characteristics of all study participants are shown in Table 1 and mean scores of various pelvic floor-related quality of life domains are presented in Table 2. 96/200 (48%) women reported psychological strain in at least 1 of the 4 pelvic floor domains while the remaining 104 women (52%) were asymptomatic.

**Table 1:** Patients’ characteristics, obstetrical and neonatologic data of the study population (mean values and SD); *n* = 200

Parameter	*n* (%)	Mean (± SD)
Age (years)		32 (± 5.7)
Current BMI (kg/m^2^)		28 (± 7.2)
BMI before pregnancy		25 (± 7.7)
Smoking	36 (18%)	
Parity		1 (± 1.2)
Family history		
Pos.	51 (26%)	
Neg.	149 (74%)	
Multiple pregnancy	22 (11%)	
Mode of delivery		
SVD	97 (49%)	
Vaginal-operative	14 (7%)	
Cesarean section	89 (44%)	
Fetal weight		3174 (± 617.4)
Gestational age (at recruitment time)		26 (± 12.6)

*SD*  standard deviation, *SVD* spontaneous vaginal delivery

**Table 2:** Mean values (SD) of pelvic floor-related quality of life domains in all cases as well as in the group with and without pelvic floor complaints

	All (*n* = 200)	PFD (*n* = 96)	Asymptomatic (*n* = 104)	*p* value
Domains	Mean (± SD)	Mean (± SD)	Mean (± SD)	
Bladder	1.52 (± 1.01)	2.01 (± 1.07)	1.07 (± 0.68)	0.0001*
Bowel	1.39 (± 0.94)	1.75 (± 1.04)	1.06 (± 0.68)	0.002 *
Prolapse	0.93 (± 1.64)	1.74 (± 1.98)	0.15 (± 0.49)	0.0001*
Sexual function	0.84 (± 0.99)	1.15 (± 1.17)	0.54 (± 0.66)	0.0001*
In all	4.68 (± 3.25)	6.63 (± 3.41)	1.98 (± 1.23)	0.001*

*SD*  standard deviation

*Significant, *p* < 0.05

### Clinical differences between the group with pelvic floor symptoms and women without any complaints

Women with PFDs showed a significant higher BMI (mean: 29.9 [± 7.3] vs. 26.5 [± 6.8]; *p* = 0.001) as well as a more frequent positive family history (43/96 [45%] vs. 8/104 [8%]; *p* = 0.0001) compared to asymptomatic women. Furthermore, patients with multiple pregnancy suffered significantly more frequent from pelvic floor symptoms compared to women with singleton pregnancy (20/96 [21%] vs. 2/104 [2%]; *p* = 0.0001). Regarding age, parity, smoking, mode of delivery and use of episiotomy, no statistically significant differences could be observed between the two groups (*p* > 0.05).

### Correlations between clinical parameters and condition-specific pelvic floor domains in women suffering from any PFD

The mean scores of the sub domains bladder, bowel, prolapse and sexual function are shown in Table 2. Mean bladder score was significantly higher in women interviewed during the third trimester of their pregnancy compared to those recruited during their first trimester (1.83 ± 1.09 vs. 1.21 ± 0.79; *p* = 0.0001). Likewise, mean prolapse scores were significantly higher in third compared to first trimester (2.18 ± 2.86 vs. 0.57 ± 1.59; *p* = 0.0001), indicating symptoms seem to worsen at the end of pregnancy. Furthermore, a statistically significant positive correlation could be observed between BMI, smoking and mean bladder score (correlation coefficient = 0.0001 and 0.005) as well as mean prolapse score (correlation coefficient = 0.0001 and 0.002), signifying more symptom bother from bladder and prolapse in female smokers with high BMI. Additionally, a significant inverse correlation was detected between mean bowel score and parity (correlation coefficient = 0.008), demonstrating more discomfort from bowel function with increasing parity.

### Multiple logistic regression analysis

Multiple logistic regression analysis was conducted to define the impact of different variables to PFDs. The presence of PFD was defined as the dependent variable. Independent variables included in the model were age, BMI, parity, smoking, multiple pregnancy and family history. After multiple logistic regression analysis, the strongest factors associated with PFD were high BMI, positive family history and multiple pregnancy (*p* = 0.017; *p* = 0.0001; *p* = 0.0001) (Table 3).

**Table 3:** Multivariate logistic regression analysis with the presence of a PFD as the dependent variable and clinical characteristics as independent variables

Parameter	OR	95% CI	*p* value
Age	0.014	0.955–1.077	0.651
BMI	0.073	1.013–1.143	0.017*
Smoking	0.140	0.461–2.860	0.764
Parity	0.175	0.905–1.569	0.211
Multiple pregnancy	2.978	0.011–0.240	0.0001*
Family history	2.235	0.044–0.260	0.0001*

*OR* odds ratio, *CI* confidence interval

*Statistically significant

## Discussion

Pelvic floor disorders are not only a major health problem, but also affect the quality of life of a woman significantly during all stages of her life—from pregnancy, early postpartum period until menopause [1]. However, despite their significant health and economic impact, little progress has been made in the prevention of pelvic floor disorders and counseling on pelvic floor function is mostly not part of routine practice during pregnancy care [6, 11].

The aim of our study was to evaluate the prevalence of PFDs in a cohort of Austrian women either during early or late pregnancy. Furthermore, we searched for clinical risk factors which correlate with the occurrence of PFDs during pregnancy in our population.

## Main findings

Our data observed that 48% of women reported significant psychological strain in at least one of the four pelvic floor domains during their pregnancy. Concerned women showed a significant higher BMI, a more frequent positive family history and a higher rate of multiple pregnancies compared to asymptomatic women (*p* < 0.05). In summary, obesity, positive family history and multiple pregnancies remained independently associated with pelvic floor symptoms during pregnancy (*p* < 0.05).

### Comparison with the literature

The reported prevalence of PFD varies widely in the literature both during and after pregnancy [2–4, 12, 13]. The observed prevalence of 48% of these conditions in our study population confirms the high prevalence and outlines the importance of this topic. Reported bladder and prolapse scores during late pregnancy were inferior to scores during early pregnancy. Such differences between gestational periods are well described in literature, indicating that PFDs seem to worsen with advanced pregnancy. Yohay and colleagues reported significant differences of the majority of the questionnaire items (PFDI-20) between late gestation and postpartum period [1]. Similarly, several authors observed that the prevalence of UI, e.g., is maximal not postpartum, but rather during second half of the pregnancy [2, 3]. In our opinion, the high prevalence of PFDs during pregnancy, observed in our study population, illustrates clearly the importance of this health problem. Our findings underline that there is a strong need to create awareness of this issue among doctors as well as among patients and adequate counseling and prevention should be a permanent part during pregnancy care. Liu et al. demonstrated in a recent survey that PFD is a prevalent condition also in young Asian pregnant women, but their general knowledge level on pelvic floor disorders was found to very be low [14].

Furthermore, our results stated that pregnant women with pelvic floor symptoms had a significant higher BMI, a more frequent positive family history and multiple pregnancies compared to asymptomatic women. It is well known that the cause of PFD is multifactorial, including many non-obstetrical risk factors such as obesity, menopause, heavy lifting and so on [15, 16]. In accordance to our study, obesity is a well-known significant risk factor for various PFDs [17]. Numerous trials have demonstrated an association between obesity and UI, POP and colorectal symptoms [17, 18]. As obesity remained an independent risk factor for pelvic floor symptoms in our study population, one may hypothesize that weight control as well as an adequate weight gain during pregnancy is an important part in counseling and prevention.

Interestingly, the percentage of twin (multiple) pregnancies was significantly higher in our group with pelvic floor symptoms and we identified multiple pregnancies as an independent risk factor for PFDs. In view of the fact due to assisted reproduction that the prevalence of twin pregnancies significantly rose in the past 30 years, this finding is of special clinical importance, creating a pressing need to study the impact of twin pregnancy on the pelvic floor. Kubotani et al. also showed that 3D ultrasound measurements of the hiatus as well as the sagittal and corona diameters of the levator ani are higher in twin than singleton pregnancies [19]. In general, the literature investigating the relationship between multiple pregnancies and PF complaints is very scarce. Béchard et al. compared the impact of mode of delivery [vaginal delivery (VD) versus cesarean section (CS)] on the pelvic floor in twin primiparae at 3 and 12 months postpartum and as a secondary end point pelvic floor dysfunction. The authors summarized that mode of delivery appears to be significantly associated with POP symptoms 3 months postpartum in twin pregnancies, which regress by 12 months [20]. Due to the high percentage of multiple pregnancies within the group of PFDs, we concluded that we should also turn our attention to twin mothers to be who suffer more frequent from pelvic floor symptoms and counsel them also with regard to PFDs.

Furthermore, Milsom and colleagues reported that a positive family history (mother and/or sister) is a major risk factor for a subsequent pelvic floor dysfunction [21]. This is also in line with our findings as positive family history remained a significant risk factor.

## Strengths and limitations of the study

Our study shows several strengths including the prospective study design, the use of a validated translated questionnaire and the representative study population of a tertiary center within a university hospital setting. Furthermore, the main outcome variable of interest was subjective pelvic floor-related symptoms and also their representation on women’s quality of life. Otherwise, the authors are also aware of the limitations of the study. The recruitment phase included either first or third trimenon of pregnancy, early or late postpartum period were not included in our survey. A follow-up visit with a second interview was also not included in our study design. Due to this fact, the authors can only comment on symptoms at a certain time during pregnancy, but not on progression or regression of the disease. Long-term data with an adequate follow-up are necessary to make definitive conclusions. Furthermore, a subgroup analysis in a bigger cohort of women with multiple pregnancies should be included in further research projects.

## Summary

In conclusion, our results demonstrate that pelvic floor-related quality of life is a prevalent as well as a relevant condition during whole pregnancy, strongly influenced by the mother’s-to-be weight, family history and expected multiple pregnancy. Adequate counseling and prevention should be a permanent part during pregnancy care and especially women with the above-mentioned risk factors should be advised particularly with regard to pelvic floor dysfunction and prevention programs.
